# Emergence of canine adenovirus infection in mink from five provinces in Northern China

**DOI:** 10.3389/fimmu.2026.1909006

**Published:** 2026-10-28

**Authors:** Jinfeng Xu, Jinyu Hou, Sheng Qin, Wenxuan Qiu, Yichang Duan, Ben Wang, Hongling Zhang, Baishuang Yin, Yanzhu Zhu

**Affiliations:** 1 Key Lab of Preventive Veterinary Medicine in Jilin Province, College of Animal Science and Technology, Jilin Agricultural Science and Technology College, Jilin, China; 2 College of Animal Science and Technology, Heilongjiang Bayi Agricultural University, Daqing, China

**Keywords:** canine adenovirus 1, epidemiology, fiber, mink, positive rate

## Abstract

Canine adenovirus 1 (CAV-1) is the causative agent of canine infectious hepatitis and fox encephalitis. However, epidemiological information regarding CAV-1 infection in mink remains limited. From September 2022 to February 2023, a total of 540 rectal swab samples from 18 mink farms from five provinces in China were collected, and viral nucleic acids were identified, sequenced, and analyzed. The overall positive rate across mink farms was 34.00%, with that of Shandong Province being the highest (67.33%). In Shandong Province, the positive rate of mink CAV-1 in Rizhao city was 100%. According to the fiber phylogenetic tree, the fiber sequences in the cities of Qinhuangdao (042803), Rizhao (042801), and Weihai (050101) were located in the same distinct branch and were different from strains isolated from other animals. These obtained fiber sequences were under purifying selection compared with the CAV-1 fiber (AC_000003.1). Compared with the fiber sequence AP_000069.1, the mutation of the 042402-TS amino acid changed the B-cell epitope and the 3D structure. However, the binding affinity between the obtained fiber sequence and the coxsackievirus and adenovirus receptor (CAR) was not different. These results indicate that strain 042402 exhibits notable genetic divergence in the fiber fragment compared with the strains from other host species, and the prevalence of CAV-1 has been confirmed in mink. Therefore, it is important to further clarify the evolution and molecular epidemiology of CAV-1 in mink in the future.

## Introduction

Canine adenovirus 1 (CAV-1) belongs to the Adenoviridae family and the *Mastadenovirus* genus (1). CAV-1 is the pathogen of canine infectious hepatitis and fox encephalitis (2). CAV-1 infection is characterized by convulsions, acute encephalitis, hepatitis, uveitis (corneal edema, “blue eye”), and interstitial nephritis (3–5). After CAV-1 infection, a marked sinusoid congestion and hepatocyte swelling in the liver, lymphopenia in the white pulp of the spleen, and interstitial vascular congestion in the kidney were observed in fox (5).

In recent years, CAV-1 infection was found in dogs in the UK between 2008 and 2018 (6). In southern Italy, between 2017 and 2020, CAV-1 was found in six (2.1%) dead stray dogs (2). In Asturias, from 2009 to 2018, the distribution of CAV-1 was found among free-ranging wolves (*Canis lupus*) and brown bears (7). In addition, CAV-1 has also been found in red foxes (*Vulpes vulpes*) in northern Norway (8), fennec fox (*Vulpes zerda*) from Egypt (9), and Arctic foxes (*Vulpes lagopus*) from Svalbard archipelago (8). Thus, CAV-1 warrants more attention, and monitoring of the prevalence of CAV-1 remains challenging.

Mink are small carnivores belonging to the Mustelidae family. Denmark, the Netherlands, and China constitute its primary breeding regions (10). As one of the representative animals, mink play a critical role in spreading several viral diseases. In 2020, severe acute respiratory syndrome coronavirus 2 (SARS-CoV-2)-infected mink were identified on 290 out of 1,147 mink farms in Denmark (11). Moreover, an influenza (H5N1) pandemic outbreak occurred at a Spanish mink farm (12). In our previous research, CAV-1 was detected in mink, and it was different from the strains found in foxes (13). These findings highlight the need for prospective monitoring of the temporal and geographical distributions and the positive rates of the currently circulating CAV-1 strains in mink.

In this study, from September 2022 to February 2023, the presence of CAV-1 in mink from the provinces of Shandong, Liaoning, Hebei, Jilin, and Heilongjiang in northern China was explored to clarify the epidemiological status of CAV-1.

## Materials and methods

### Ethics statement

All experimental procedures were reviewed and approved by the IACUC of Jilin Agricultural Science and Technology College and were conducted according to institutional guidelines. The approved research protocol number is LLSC202301010.

### Sample collection and viral nucleic acid extraction

The criteria for random sample collection were as follows. The farms housed both mink and foxes. The mink had not been vaccinated with the CAV-2 live vaccine. Mink present with icterus of the conjunctivae, without any other clinical signs. Dogs are kept on the farm. In addition, there were no infectious diseases reported on the farms during the sampling period. Mink farms located in different regions of these provinces were selected. All sampled mink were 1 year old.

From September 2022 to February 2023, rectal swabs were collected from 540 1-year-old mink from 18 mink farms. Rectal swab samples with severe hemolysis, extensive putrefaction, or storage at room temperature for more than 6 h were excluded. Five farms were located in Linyi, Zhucheng, Qingdao, Rizhao, and Weihai, in Shandong Province. Two farms were in Tangshan and Qinhuangdao, in Hebei Province. Five farms were in Wafangdian, Chengzitan, Jinzhou, Pikou, and Zhuanghe, in Liaoning Province. One farm was in Changchun, Jilin Province. Five farms were in Yichun and Shuangcheng, in Heilongjiang Province, China. A total of 30 samples were collected from each farm (20 males and 10 females). Sample DNA was extracted according to the kit instructions (ER201-02; TransGen Biotech, Beijing, China) and stored at −20°C.

### Identification of CAV-1 in mink

To discriminate between CAV-1 (508 bp) and CAV-2 (1,030 bp), the CAV-1 identification primers HA1 (5′-CGCGCTGAACATACTACTACTTTGTC-3′) and HA2 (3′-CCTAGAGCACTTCCGTCCGCTT-5′) were used (14). PCR amplification was performed on DNA samples of 540 mink rectal swabs with 2× EasyTaq PCR SuperMix (AS111, TransGen). The PCR conditions were as follows: initial denaturation at 95°C for 1 min, followed by 35 cycles of denaturation at 95°C for 15 s, annealing at 59°C for 30 s, extension at 72°C for 45 s, and a final extension at 72°C for 7 min. Positive (CAV-1 DNA) and negative controls (double-distilled water, ddH_2_O) were included in the PCR assays. All PCR products were electrophoresed on 1% agarose gel for 35 min, and the results were observed with a gel imaging machine (Bio-Rad, Hercules, CA, USA). Finally, the positive rates were calculated.

### Construction of CAV-1 fiber plasmid

SnapGene software (Dotmatics, Boston, MA, USA) was used to design the primers for the CAV-1 fiber. Fiber primers (both 956 bp) F (5′-CCGGGTCTCACCGTACTAAACG-3′) and R (3′-GCAGCGCCAGCTAGTTGTT-5′) were used to amplify the HA1/HA2-positive samples. PCR was performed using 2× EasyTaq PCR SuperMix (AS111, TransGen), as follows: 35 cycles of 95°C pre-denaturation for 1 min, 95°C denaturation for 15 s, 60°C annealing for 30 s, 72°C extension for 1 min, and 72°C final extension for 7 min. Both positive (CAV-1 DNA) and negative controls (ddH_2_O) were included in the PCR assays. All products were electrophoresed on 1% agarose gel for 35 min, and the results were observed with a gel imaging machine (Bio-Rad).

The target product was extracted using a gel recovery kit (D2500-01; OMEGA, Norcross, GA, USA), connected to the pMD18-T vector, and was transformed into DH5α competent cells. The transformation mixture was plated onto agar plates and then incubated at 37°C overnight (12–14 h). Single colonies were picked and cultured in Luria–Bertani (LB) broth at 37°C with shaking at 220 rpm for 12–14 h. The plasmid was then extracted using the kit (D6943-02, OMEGA) and stored at −20°C.

### Nucleotide identity and sequence analysis

The plasmid was sent for sequencing to Shanghai Sangon Biotechnology Co., Ltd. (Shanghai, China). The fiber sequences obtained in this study were analyzed and compared with public canine fiber sequences (AC_000003.1) using the Basic Local Alignment Search Tool (BLAST) (15). The nucleotide mutations in the obtained sequence were analyzed using MEGA (Sudhir Kumar, version 11) and Lasergene (version 17.6; DNAStar, Madison, WI, USA) (16).

### Phylogenetic analysis

Multiple sequence alignments were performed using MAFFT with the auto strategy (Kazutaka Katoh, version 7.526) (17). A preliminary neighbor-joining tree was constructed under the best-fit substitution model to provide the starting topologies for subsequent maximum likelihood (ML) analyses. The phylogenetic trees were reconstructed using IQ-TREE (Bui Quang Minh, version 2.1.3) (18) with the ML method. ML inference aims to identify the tree topology and branch lengths that maximize the probability of observing the sequence data under a specified evolutionary model (19). IQ-TREE implements an efficient stochastic search algorithm that combines hill-climbing and perturbation strategies to thoroughly explore the tree space and convergence on the optimal topology (18). The best-fit evolutionary model for each dataset was automatically selected using ModelFinder implemented in IQ-TREE based on the Bayesian information criterion (BIC) (20). Bootstrap support values were calculated using 1,000 replicates to assess the statistical reliability of the tree topologies. The bootstrap procedure generates pseudoreplicate datasets by randomly sampling the alignment columns with replacement, and the frequency with which a given clade is recovered across all replicates is reported as the bootstrap support value (21). Values ≥80% were considered strong support and were indicated at the corresponding nodes. All trees were visualized with ggtree (Guangchuang Yu, v3.16) (22).

### Amino acid mutation analysis

The nucleotide sequences of each virus were translated into amino acid sequences using the Biopython package (version 1.79) with appropriate reading frames. The amino acid mutation analyses of the 13 obtained fiber sequences were compared with the fiber protein AP_000069.1 using MEGA (Sudhir Kumar, version 11).

### Prediction of the B-cell epitope, 3D structures, and binding affinity of the obtained fiber sequences

Synonymous and non-synonymous substitutions were analyzed with MEGA (Sudhir Kumar, version 11). B-cell epitopes were predicted using the IEDB Analysis resource (https://tools.iedb.org/bcell/). Linear B-cell epitopes are typically 8–20 amino acids (aa), preferably 12–16 aa (15-mer is classic). The protein sequence of the coxsackievirus and adenovirus receptor (CAR) in ferret is found in Uniprot (https://www.uniprot.org/). The 3D structures of the obtained fiber sequence, CAV-1 fiber, and ferret CAR were predicted using Swiss-MODEL (https://swissmodel.expasy.org). Differences in the structure were compared with US-align (https://zhanggroup.org/US-align/). A TM-score >0.5 denotes identical overall fold/topology, whereas a TM-score <0.5 indicates marked divergence in global tertiary folding. When the TM-score is <0.5, the root mean square deviation (RMSD) is less informative and the TM-score serves as the main metric. RMSD quantifies the 3D backbone deviation of the aligned residues, while Seq_ID denotes sequence similarity. The binding model between the fiber and CAR was built using the HawkDock Server v2 (https://cadd.zju.edu.cn). The binding affinity was calculated with PRODIGY (https://wenmr.science.uu.nl/prodigy). A negative Δ*G* (in kilocalories per mole) means spontaneous binding. A more negative Δ*G* corresponds to stronger affinity, as follows: −5 ~ −8 kcal/mol (weak), −8 ~ −12 kcal/mol (moderate), and −12 ~ −18 kcal/mol (strong high-affinity binding). For the dissociation constant, *K*_d_ (in molar unit), a smaller *K*_d_ indicates higher affinity: micromolar indicates weak binding, nanomolar for moderately high affinity, and picomolar for extremely strong protein–protein interactions.

## Results

### Identification and positive rates of CAV-1 in mink

The PCR product was 508 bp, which was confirmed to be CAV-1 (**Figure 1A**). The positive rates in the five provinces are shown in **Figure 1C**. The positive rate of CAV-1 in Shandong Province was the highest, with a total positive rate of 67.33%. The positive rate of CAV-1 in Shandong-Rizhao was 100%. Those in Weihai, Zhucheng, Qingdao, and Linyi were also relatively high, at 76.67%, 66.67%, 46.67%, and 46.67%, respectively. The positive rate of CAV-1 in Hebei-Tangshan was 86.67%, while those in Liaoning-Jinzhou and Liaoning-Wafangdian were 70.00% and 60.00%, respectively. CAV-1 was not detected in the five farms in Heilongjiang Province (**Table 1**).

**Figure 1:**
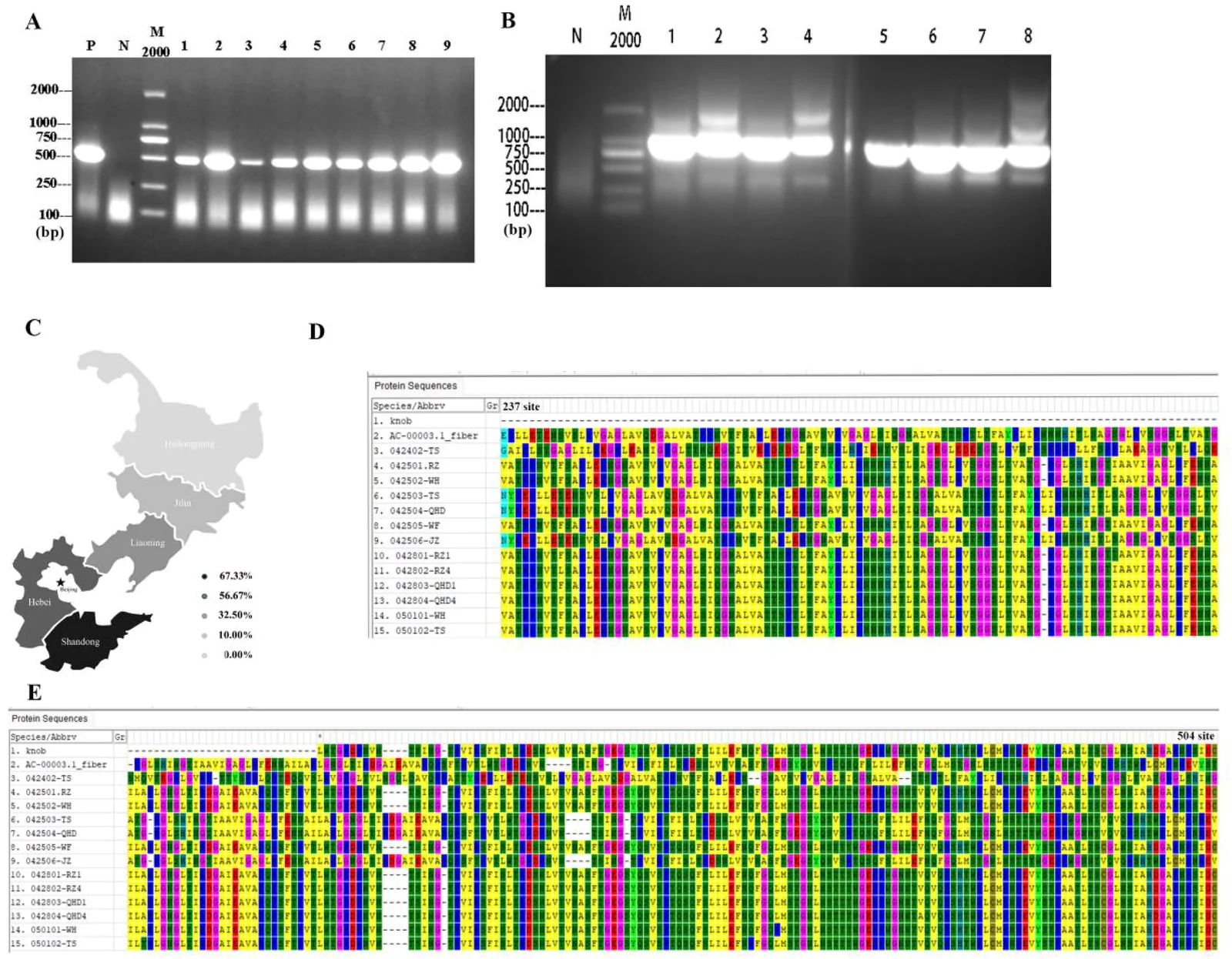
Canine adenovirus 1 (CAV-1) identification and analysis in mink. **(A)** Identification of mink CAV-1 (508 bp). *P*, positive sample; *N*, negative sample. M2000 is the marker. Numbers *1*–*9* denote the partial PCR results of CAV-1 in the rectal swabs from different farms. **(B)** Identification of mink CAV-1 (Fiber-956bp). *P*, positive sample; *N*, negative sample. M2000 is used as the marker. *1*, Shandong–Weihai; *2*, Shandong–Rizhao; *3*, Shandong–Linyi; *4*, Shandong–Zhucheng; *5*, Hebei–Tangshan; *6*, Hebei–Qinhuangdao; *7*, Liaoning–Jinzhou; *8*, Liaoning–Wafangdian. **(C)** Map of the distribution of mink CAV-1 in five provinces in China. The color changes from *dark* to *light*, indicating a decrease in the positive rate from high to low. **(D, E)** The amino acid mutations in the obtained 13 fiber sequence compared with fiber AC_000003.1.

**Table 1:** Canine adenovirus 1 (CAV-1) positive rates in mink.

Toponym	Sample size		Positive quantity		Positive rate (%)
	Male	Female	Male	Female	
Jilin-Changchun	20	10	2	1	10.00
Shandong-Linyi	20	10	7	7	46.67
Shandong-Weihai	20	10	14	9	76.67
Shandong-Zhucheng	20	10	10	10	66.67
Shandong-Qingdao	20	10	11	3	46.67
Shandong-Rizhao	20	10	20	10	100.00
Hebei-Tangshan	20	10	19	7	86.67
Hebei-Qinhuangdao	20	10	4	4	16.67
Liaoning-Wafangdian	20	10	11	7	60.00
Liaoning-Chengzitan	20	10	1	6	23.33
Liaoning-Jinzhou	20	10	12	9	70.00
Liaoning-Pikou	20	10	0	0	0.00
Liaoning-Zhuanghe	20	10	5	1	20.00
Heilongjiang-Yichun	20	10	0	0	0.00
Heilongjiang-Shuangcheng-01	20	10	0	0	0.00
Heilongjiang-Shuangcheng-02	20	10	0	0	0.00
Heilongjiang-Shuangcheng-03	20	10	0	0	0.00
Heilongjiang-Shuangcheng-04	20	10	0	0	0.00
Total positive rate (%)					34.00

### Sequence analysis of CAV-1 fiber

CAV-1 fiber was amplified from the CAV-1-positive samples (**Figure 1B**), and plasmids were constructed for sequencing. A total of 13 CAV-1 fibers obtained from mink were compared with CAV-1 fiber AC_000003.1. As shown in **Supplementary Table S1**, mutations were found in the sequence at positions 26541(A042803G), 26619(T042802C), 26666(A050102C), 26741(A-all-G), 26783(C042803T), 26851(T042801C), 26870(A042801G), 26901(G050102A), 27023(T042801C), 27166(A050102C), 27178(T050101C), 27244(T042804C), and 27347(A042802G). Compared with the amino acid sequence of the fiber protein AP_000069.1, amino acid mutations were found in the obtained fiber sequences. More mutations were found in the 042402-TS fiber sequence (**Figures 1D, E**).

### Phylogenetic tree of CAV-1 fiber

As shown in **Figure 2**, the strains of CAV-1 in mink in the cities of Rizhao (042801), Qinhuangdao (042803), Tangshan (042402), and Weihai (050101) were clustered into a distinct Chinese branch. These strains exhibited notable genetic divergence in the fiber fragment compared with the CAV-1 strains from dogs, wolves, foxes, and bears. The other strains from dogs, wolves, foxes, and bears were clustered into two Chinese groups. These two groups clustered with the reference CAV-1 strains. The CAV-1 strains in mink from the cities of Jinzhou (042506), Qinhuangdao (042504), and Tangshan (042503) clustered into a distinct branch. The CAV-1 strains in mink from the cities of the Rizhao (042802 and 042501), Qinhuangdao (042804), Tangshan (050102), Weihai (042502), and Wafangdian (042505) also clustered into a distinct branch.

**Figure 2:**
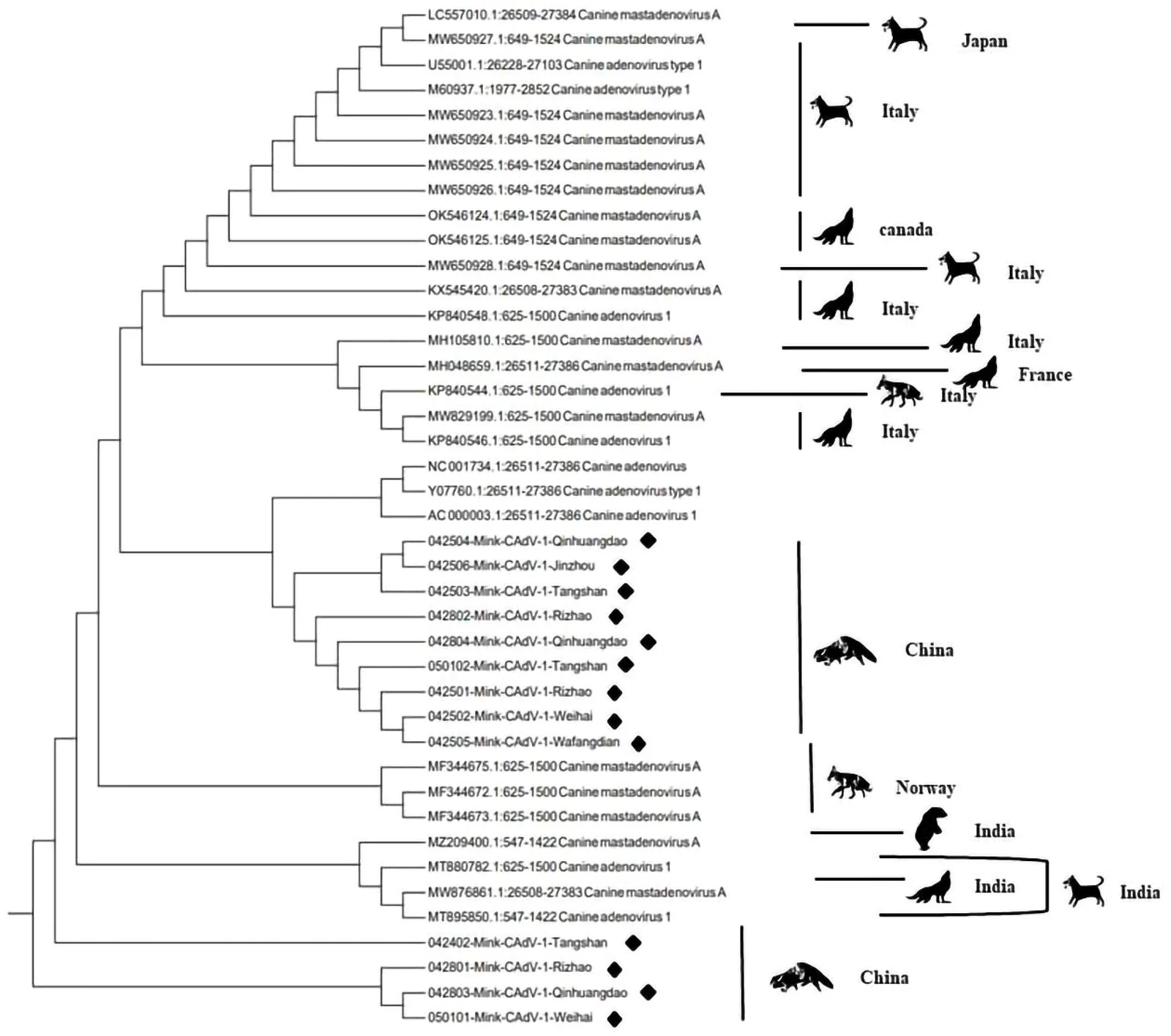
Phylogenetic tree of the canine adenovirus 1 (CAV-1) fiber. The *black block* annotation represents the sequence measured in this experiment. The GenBank accession numbers were as follows: 042505(OR215494), 050101(OR146640), 050102(OR167626), 042402TS(PZ688189), 042501RZ(PZ688190), 042502WH(PZ688191), 042503TS(PZ688192), 042504QHD(PZ688193), 042506JZ(PZ688194), 042801RZ1(PZ688195), 042802RZ4(PZ688196), 042803-QHD1(PZ688197), and 042804-QHD4(PZ688198).

### B-cell epitope, 3D structure, and binding affinity of the obtained fiber sequences

Synonymous and non-synonymous substitutions were analyzed, which showed a *t* value of −0.201 and a *p*-value of 1.00 using the *t*-test for positive selection. These results indicate that these sequences were under purifying selection compared with the CAV-1 fiber AC_000003.1. Compared with the fiber sequence AP_000069.1, the mutation of the amino acid changed the B-cell epitope in the obtained fiber sequence, in particular the 042402-TS amino acid sequence. Compared with the fiber protein AC_000003.1, the fiber3D structures of the fibers from 042402-TS, 042801-RZ1, 042804-QHD4, 050101-WH, and 050102-TS showed differences compared with that of the CAV-1 fiber protein AP_000069.1 according to the TM-score, RMSD, and Seq_ID results. The 3D structure of the fiber from strain 042402-TS showed substantial divergence. Compared with the fiber protein AP_000069.1, the binding affinity between the obtained fiber sequence and CAR was not different according to the Δ*G* and *K*_d_ results (**Table 2**).

**Table 2:** Prediction of the B-cell epitope, 3D structures, and binding affinity of the obtained fiber sequences.

Sequence	B-cell epitope	Structure difference			Binding affinity	
		TM-score	RMSD	Seq_ID (%)	Δ*G *(kcal/mol)	*K*_d_ (pM)
042402-TS	GLTESSPG	0.329	0.63A	21.3	−15.3	5.5e−12
042501-RZ	VQDGALVA, APQPSFTP, DPNVNTSIN, GSYQSVSPTQ	1.000	0.00A	100	−15.0	1e−11
042502-WH	VQDGALVA, APQPSFTP, DPNVNTSIN, GSYQSVSPTQ	1.00	0.00A	100	−15.0	1e−11
042503-TS	GALVATPPN, SVRVGAGLSI, APQPSFTP, DPNVNTSI, GSYQSVSPTQ	1.00	0.00A	100	−15.0	1e−11
042504-QHD	GALVATPPN, SVRVGAGLSI, APQPSFTP, DPNVNTSI, GSYQSVSPTQ	1.00	0.00A	100	−15.0	1e−11
042505-WF	SVRVGAGLSI, APQPSFTP, DPNVNTSIN, GSYQSVSPTQ	1.00	0.00A	100	−15.0	1e−11
042506-JZ	GALVATPPN, SVRVGAGLSI, APQPSFTP, DPNVNTSI, GSYQSVSPTQ	1.00	0.00A	100	−15.0	1e−11
042801-RZ1	VQDGALVAT, SYQSVSPTQ, MPNREVYSTPAATLT	0.99897	0.17	99.5	−15.4	4.7e−12
042802-RZ4	VSVRVGAGLS, APQPSFTPV, GPDPNVNTSIN, SYQSVSPTQ, MPNREVYSTPAATLTSC	1.00	0.00A	100	−15.0	1e−11
042803-QHD1	VSVRVGAGL, PTLTFAYPLIKNN, LTIRDGAIEAVAPQPSFTP, PDPNVNTSI, GSYQSVSPTQ, MPNREVYSTPAATLT	1.00	0.00A	100	−15.0	1e−11
042804-QHD4	VQDGALVATP, VAPQPSFTP, DPNVNTSIN, SYQSVSPTQ	0.99580	0.34	99.5	−14.9	1.1e−11
050101-WH	DGALVATP, SVRVGAGLS, PQPSFTPV, PDPNVNTSI, SYQSVSPTQ, MPNREVYSTPAATLTS	0.99564	0.41	99.5	−14.9	1.3e−11
050102-TS	VSVRVGAGLSI, APQPSFTPV, PDPNVNTSI, SYQSVSPTQ, MPNREVYSTPAATLTSC	0.99998	0.02	98.9	−15.4	5.2e−12
AC_000003.1	SVRVGAGLSI, APQPSFTP, DPNVNTSIN, GSYQSVSPTQ	1.00	0.00A	100	−14.9	1.1e−11

For strain 042402-TS, the TM-score, root mean square deviation (RMSD), and sequence similarity (Seq_ID) were calculated on spatially aligned fragments (61 residues) rather than the full-length sequence. A TM-score <0.5 indicates divergence in global topology; in such a case, the RMSD only reflects deviation of the aligned fragment and cannot represent the full-length structure.

## Discussion

CAV-1 has infected various mammals and has attracted considerable attention worldwide (1). Shandong, Liaoning, and Hebei are the major provinces in China for mink farming. This study focused on the circulating trend of CAV-1 in the mink in the provinces of Shandong, Hebei, Liaoning, Jilin, and Heilongjiang in northern China from September 2022 to February 2023.

Rectal swabs could be efficiently collected and retained as samples as they are not prone to shedding or contamination. The operation is simple and could be easily performed by veterinarians. They can be used to collect samples from various parts of an animal’s body to meet different testing requirements. The material is safe and non-toxic, causing no irritation or harm to animals. After sampling, the swab head could be easily placed into the preservative fluid in the storage tube for safe transport and storage. Thus, rectal swabs were chosen to collect samples from mink. To our knowledge, systematic studies comparing the diagnostic sensitivity of different sample types for CAV-1 detection in mink are currently lacking in the literature. In the absence of such comparative data, rectal swabs represented a pragmatic compromise between diagnostic yield and field applicability. Oropharyngeal swabs, while biologically more intuitive for a respiratory pathogen, require trained personnel and may cause stress-induced sampling bias in mink, whereas liver tissue, the gold standard for confirming hepatotropic infection, necessitates euthanasia and is incompatible with routine epidemiological surveillance.

The positive rate of CAV-1 in Shandong Province was the highest, with a total positive rate of 67.33%. The positive rate of CAV-1 in mink from Shandong-Rizhao was 100%. Shandong Province had the most number of mink farms and is located in the coastal region. The CAV-1 in mink may be transmitted from dogs and foxes on the same farm because CAV-1 has been reported in these animals. However, there are limited data to support this hypothesis. In “Pi Kou, Liaoning Province,” the positive rate was 0.00%, which may be related to factors such as the density of animals in the farms, the ventilation conditions of the enclosures, and the source of stock. However, CAV-1 was not detected in the five mink farms in Heilongjiang. Currently, the prevention of CAV infection mainly relies on dog and fox vaccination (23). However, the prevalence of CAV-1 is still present in canine populations (8). Researchers have also detected CAV-1 in wild animals (24). As susceptible animals, dogs and foxes may harbor CAV-1 for a long time, thus acting as reservoirs (4). The circulation of CAV-1 may be caused by transmission between dogs and foxes, leading to the disease in wild animals (8). The rectal swab-based prevalence data should be interpreted with caution as a conservative estimate of the true infection rates. There is an urgent need for future comparative studies to evaluate diagnostic sensitivity across oropharyngeal, nasal, rectal, and tissue specimens in mink, and we have tempered our conclusions regarding CAV-1 epidemiology to reflect the uncertainty introduced by the sampling methodology.

Adenoviruses are highly prevalent pathogens that can cause different levels of infectivity, viral titers, and pathogenicity in different species (25). The family Adenoviridae is divided into five genera, each corresponding to an independent evolutionary lineage that supposedly co-evolved with its respective vertebrate hosts. However, the adenovirus in mink is elusive. According to the fiber phylogenetic tree, the CAV-1 strains in mink were clustered into three groups. The nine strains in mink are closely related to the canine strains. However, the other four strains (042402, 042801, 042803, and 050101) were different from the canine strains. The CAV-1 strains in Tangshan (042402), Rizhao (042801), Qinhuangdao (042803), and Weihai (050101) may be different, and virulence and local spread should be explored for the circulating virus strain in the future. Although the observed variability of the fiber sequences was low, the frequency and geographical distribution of some variants suggest a recent positive selection of favorable amino acid changes. Further experiment is necessary to confirm this hypothesis.

Mutations and single nucleotide substitutions in CAV-1 fiber may cause differences in the disease pathogenesis. The results of the synonymous and non-synonymous substitutions in the *t*-test for positive selection indicated that both synonymous and non-synonymous substitutions occurred in the examined sequences. However, the non-synonymous substitution rate was lower than the synonymous substitution rate, suggesting purifying selection rather than positive selection. Under maternal or host neutralizing antibodies, natural selective pressure may drive these additional changes in the fiber sequence. The distribution of the amino acid changes may confer selective advantages to CAV-1. The strains from mink exhibited genetic differences from those from dogs. With the implementation of new vaccines, it is hypothesized that immunity induced by CAV-1 vaccination may impose new selection pressures on the CAV-1 circulating strain and create the possibility of new viral lineages. Compared with the amino acid sequence of the fiber protein AP_000069.1, mutations were found in the obtained fiber sequences. Certain changes in the CAV-1 fiber may need to obtain the whole sequence of the fiber protein to explain the absence of clinical outbreaks in mink. These phenotypic differences between the CAV-1 lineages may reflect their differences in the fiber protein. Our prediction results also showed that the mutations changed the B-cell epitopes of the obtained fiber sequence. The 042402 strain had a different epitope compared with the CAV-1 fiber protein AP_000069.1, indicating that it is special. The antigenic site changes may alter viral antigenicity to vaccines and affect their susceptibility to monoclonal antibodies (mAbs). Further evidence is vital to demonstrate this hypothesis. It would be important to further study the implications of these mutations in the transmission of the virus and the alteration of its pathogenicity. Compared with the CAV-1 fiber protein AP_000069.1, the 3D structure of 042402 differed considerably, while those of 042801-RZ1, 042804-QHD4, 050101-WH, and 050102-TS showed slight differences. The other sequences were the same as that of the CAV-1 fiber protein AP_000069.1. For strain 042402-TS, the TM-score, RMSD, and Seq_ID were calculated on spatially aligned fragments (61 residues) rather than the full-length sequence. A TM-score <0.5 indicates divergence in global topology, and in such a case, the RMSD only reflects deviation of the aligned fragments and cannot represent the full-length structure. The knob of CAV-1 binds to the CAR. The knob protein was located at 366–538 aa. Notably, the mutation of the amino acid clustered at the knob domains, suggesting adaptive evolution in the receptor-binding region. However, according to our binding affinity results, there were no differences between the obtained fiber sequences and the CAV-1 fiber protein AP_000069.1. This indicates that the amino acid mutations changed the B-cell epitope and the 3D structure, but did not affect the binding affinity activity of the CAR. In the future, experiments are essential to confirm the binding affinity activity of the circulating CAV-1 strains.

In conclusion, this study characterized the CAV-1 strains circulating in northern Chinese mink farms from September 2022 to February 2023. The overall positive rate in mink was 34.00%, with that in Shandong Province being the highest (67.33%). Compared with the CAV-1 strains from other host species, the fiber gene fragment in strain 042402 from mink showed significant genetic divergence. It is important to clarify the evolution and molecular epidemiology of CAV-1 in mink. These findings suggest that mink may be involved in the CAV-1 transmission chain among carnivores, and further studies are required to clarify their role in cross-species transmission.

## Data Availability

The original contributions presented in the study are included in the article/**Supplementary Material**. Further inquiries can be directed to the corresponding authors.
