# Supplementary material for: Emergence of canine adenovirus infection in mink from five provinces in Northern China
Source: Front Immunol. 2026 Oct 28;17:1909006. doi: 10.3389/fimmu.2026.1909006 (PMC13751980; doi:10.3389/fimmu.2026.1909006)
Supplement: Supplementary file 1 [file Table1.docx]

**Table 1S CAV-1 *fiber* mutation sites.**

| Sequence  /Base position | 26541 | 26619 | 26666 | 26741 | 26783 | 26851 | 26870 | 26901 | 27023 | 27166 | 27178 | 27244 | 27347 |
| --- | --- | --- | --- | --- | --- | --- | --- | --- | --- | --- | --- | --- | --- |
| AC_000003.1 | A | T | A | A | C | T | A | G | T | A | T | T | A |
| 042402-TS | - | - | - | G | - | - | - | - | - | - | - | - | - |
| 042501-RZ | - | - | - | G | - | - | - | - | - | - | - | - | - |
| 042502-WH | - | - | - | G | - | - | - | - | - | - | - | - | - |
| 042503-TS | - | - | - | G | - | - | - | - | - | - | - | - | - |
| 042504-QHD | - | - | - | G | - | - | - | - | - | - | - | - | - |
| 042505-WF | - | - | - | G | - | - | - | - | - | - | - | - | - |
| 042506-JZ | - | - | - | G | - | - | - | - | - | - | - | - | - |
| 042801-RZ1 | - | - | - | G | - | C | G | - | C | - | - | - | - |
| 042802-RZ4 | - | C | - | G | - | - | - | - | - | - | - | - | G |
| 042803-QHD1 | G | - | - | G | T | - | - | - | - | - | - | - | - |
| 042804-QHD4 | - | - | - | G | - | - | - | - | - | - | - | C | - |
| 050101-WH | - | - |  | G | - | - | - | - | - | - | C | - | - |
| 050102-TS | - | - | C | G | - | - | - | A | - | C | - | - | - |
